# A conditional process analysis of general self-esteem in student-athletes in lower secondary sport schools

**DOI:** 10.3389/fpsyg.2026.1771557

**Published:** 2026-03-06

**Authors:** Siv Gjesdal, Jan Åge Kristensen, Milla Saarinen, Christian Thue Bjørndal

**Affiliations:** 1 The Child and Youth Sport Research Centre, Norwegian School of Sport Sciences, Oslo, Norway; 2 Department of Sport and Social Sciences, Norwegian School of Sport Sciences, Oslo, Norway; 3 Department of Sport Science and Physical Education, Faculty of Health and Sport Sciences, University of Agder, Kristiansand, Norway; 4 Faculty of Arts and Education, University of Stavanger, Stavanger, Norway

**Keywords:** dual career, motivation, psychosocial factors, talent development, youth sport

## Abstract

**Objective:**

Specialised sport schools at the lower secondary level aim to support the holistic development of young athletes. Consequently, understanding how to influence student-athletes’ self-perceptions both inside and outside of the sporting context is of interest. Grounded in Achievement goal theory, this study examined whether achievement goal orientations were related to self-esteem, both directly and indirectly through perceived sport performance. Additionally, we investigated whether athletic identity moderated the indirect relationship between achievement goal orientations and self-esteem.

**Methods:**

The sample comprised 579 student-athletes aged 12 to 16 years (Mage = 13.93; SD = 0.85), recruited from seven Norwegian lower secondary sport schools. All participants responded to items concerning their general self-esteem, goal orientations, perceived performance and athletic identity.

**Results:**

Regression analyses revealed that athletes’ achievement goal orientations were directly and indirectly (via perceived performance) related to their self-esteem. However, athletic identity did not moderate the indirect relationship.

**Conclusion:**

Overall, the findings suggest that student-athletes who view success as doing their very best tend to be more satisfied with their own sport performance and experience higher self-esteem. Conversely, athletes who view success as being the best are generally less satisfied with their performance and report lower self-esteem. Notably, these relationships do not appear to be stronger in athletes with a stronger identification with their athlete role.

## 1 Introduction

Specialized sport schools at the lower secondary level are becoming increasingly popular in Norway (Lovdata, 2023), offering an alternative to regular secondary schools. Upper secondary school refers to the stage of education that comes after elementary or primary school, aimed at students between the ages of 13 and 16 years of age. While student-athletes in these schools meet the same academic requirements as their peers, they benefit from a more flexible class schedule and structured sports training during school hours, complementing their club-based training and competition. Admission is highly competitive, requiring the applicants to undergo physical aptitude tests and assessment of their sports performance. The stated aim of these schools is to promote young athletes’ well-being, and prepare them for a career in sport and for life beyond their athletic pursuits (Stambulova et al., 2024; Stambulova and Wylleman, 2019). However, student-athletes at both the lower and upper secondary school level face a unique set of challenges, including performance pressure, high levels of burnout and demanding training and competition schedules (Saarinen et al., 2024; Thompson et al., 2022; Kavoura and Ryba, 2020). Moreover, the growing popularity of these schools, combined with a limited number of spots, has made admission highly competitive (Øydna et al., 2024). These stressors have led researchers to emphasize the importance of identifying potential protective factors that support young student-athletes’ psychological well-being and overall development (Nikander et al., 2022; Sorkkila et al., 2020; Ryba et al., 2016).

One such factor could be general self-esteem, which has long been regarded as a key psychological factor influencing various markers of a good life, including greater initiative in life, a healthier lifestyle, life satisfaction, and happiness (Baumeister et al., 2003; Asgeirsdottir and Sigfusdottir, 2021). It is commonly defined as an evaluative component of self-perception, reflecting affective appraisals of one’s worth and value (Rosenberg, 1989). In the context of sport, scholars suggest that high self-esteem can promote positive outcomes, influencing athletes’ engagement and long-term investment in their sport (Asgeirsdottir and Sigfusdottir, 2021; Baumeister et al., 2003). Conversely, low self-esteem has been associated with an increased risk of mental illness symptoms, such as anxiety, depression and burnout (Edison et al., 2021; Markati et al., 2019). Moreover, a recent study of student-athletes in upper secondary sport schools showed that self-esteem levels varied among individuals and were related to athletic career adaptability over time (Nikander et al., 2022). Similarly, Saarinen et al. (2025b) reported that student-athletes with low self-esteem were more likely to experience symptoms of sport and school burnout.

Ferkany (2008) emphasized that self-esteem should play a central role in education, advocating for schools to actively implement strategies that foster self-esteem in their students. In the context of sport schools, which aim to support the holistic development of young athletes (Stambulova et al., 2024; Stambulova and Wylleman, 2019), understanding the factors that influence self-esteem becomes particularly pertinent. Research has demonstrated that the self-esteem of adult student-athletes is shaped by a variety of factors, including health-related behaviours (Papale et al., 2025). Additionally, studies suggest that while self-esteem tends to remain relatively stable during adolescence (Birkeland et al., 2012; Trzesniewski et al., 2003), it is still susceptible to the influence of life events, personal experiences, and the surrounding psychosocial environment (Birkeland et al., 2012). In this paper, we will focus on individual factors, specifically achievement goal orientations, as a key lens through which to understand self-esteem development.

To examine potential antecedents of self-esteem in student-athletes attending lower secondary sport schools, we relied on Achievement goal theory (AGT; Nicholls, 1984) as the theoretical framework. According to Nicholls (1984), individuals in achievement domains, such as sport, participate primarily to demonstrate competence. However, competence can be defined in different ways depending on an individual’s goal orientation. Specifically, task goal-oriented individuals view competence as synonymous with improvement, effort, learning, and task mastery. In contrast, ego goal-oriented individuals equate competence with normative ability, emphasizing superior ability and outperforming others (Nicholls, 1989; Nicholls, 1984).

From an AGT perceptive, different conceptions of competence are relevant to self-esteem, as competence is considered a key component of general self-esteem. How individuals define and experience competence will influence their self-esteem levels (Kavussanu and Harnisch, 2000). Several studies have examined the relationship between achievement goal orientations and self-esteem, with results indicating that individual differences in task and ego orientation for sport may have important implications for general self-esteem (e.g., Chen et al., 2018; Kavussanu and Harnisch, 2000). Specifically, task goal-oriented athletes, who seek to demonstrate competence by personal progress and skill development, may experience more positive self-perceptions compared to ego-oriented athletes, who derive a sense of competence by demonstrating superiority over others. Given the increasing number of student-athletes in specialized sport schools and the potential psychological challenges they face, examining these relationships within this population is particularly relevant. However, limited research has explored these dynamics in student-athletes at the lower secondary level, highlighting the need for further investigation (Saarinen et al., 2025a).

In a study of athletes in the same age group as student-athletes in lower secondary schools, Gjesdal et al. (2017) showed that the relationship between goal orientation for sport and general self-esteem was mediated by perceptions of competence, defined as how athletes evaluated their own ability in sport. In the context of sport schools, self-perceptions are likely reflected through perceived sport performance, making this an especially relevant aspect. First, lower secondary schools explicitly aim to develop the sport performance of their student-athletes (Kårhus, 2019), positioning them as key players in Norway’s talent development pathway (Kristiansen and Houlihan, 2017). Second, these student-athletes are generally highly motivated for sport (Gjesdal et al., in review) and high performing (Skrubbeltrang et al., 2020). Given the emphasis on sport performance in this setting, perceived performance is likely to mediate the relationship between goal orientation and self-esteem.

When examining self-esteem, perceived performance is more critical than objective performance. Perceived performance, or subjective performance, is viewed as multidimensional, encompassing facets such as performance, improvement, and goal achievement, and is believed to impact affective tendencies (Riemer and Chelladurai, 1998). Unlike objective outcomes, which can be influenced by chance and external factors such as injury, maturation or refereeing decisions, perceived performance reflects an athlete’s subjective evaluation of their own progress and achievement (Terry, 1995; Males and Kerr, 1996; Nicholls et al., 2012). Numerous studies have reported positive relationships between perceived performance and self-esteem, including in sport contexts (e.g., Baumeister et al., 2003; Fox and Lindwall, 2014).

Based on the aforementioned definition of perceived performance, two athletes with similar objective (i.e., normative) performance levels may experience differing levels of satisfaction with their performance. AGT offers an explanation for such differences. Task-goal oriented athletes are more likely to experience higher perceived performance because they employ self-referencing standards, which are more likely within their control compared to more ego-oriented standards. Simply put, these athletes are more likely to feel successful. Conversely, ego-goal oriented athletes rely on external comparisons, making it more difficult to consistently meet their own success criteria, likely leading them to be less satisfied with their performance over time. A caveat in the study of how achievement goal orientations and self-perceptions in specific domains relate to general self-esteem is the salience that specific achievement domain for each individual (Rosenberg et al., 1995). For example, positive self-perceptions within a domain that hold little importance to a person are not likely to contribute significantly to their general self-esteem. In the context of sport schools, the relationship between student-athletes’ perceived performance and their general self-esteem is likely to be moderated by their athletic identity. Defined by Brewer et al. (1993), athletic identity refers to the degree to which an individual identifies with the athlete role and how important sport is to their self-concept. Applied to this study, the extent to which experiences in sport will influence general self-perceptions may depend on how strongly student-athletes identify as athletes.

Research suggest that student-athletes generally report very high levels of athletic identity (Stambulova et al., 2015). However, recent findings indicate that this group is more heterogeneous than previously assumed, with some student-athletes placing equal emphasis on education and sport (Cartigny et al., 2021; Moazami-Goodarzi et al., 2020; Skrubbeltrang et al., 2020). This variability in athletic identity may influence how goal orientation impacts self-esteem through perceived performance. Specifically, for individuals with a stronger athletic identity, the connection between perceived performance and self-esteem may be more pronounced. As a result, these athletes may experience greater fluctuations in self-esteem based on their perceived sporting achievements, becoming more responsive to positive outcomes but also more vulnerable to setbacks. Therefore, a key objective of this study was to address the “for whom” question by investigating whether the relationships between goal orientation, perceived performance, and self-esteem are stronger among athletes with higher athletic identity.

**Figure 1:**
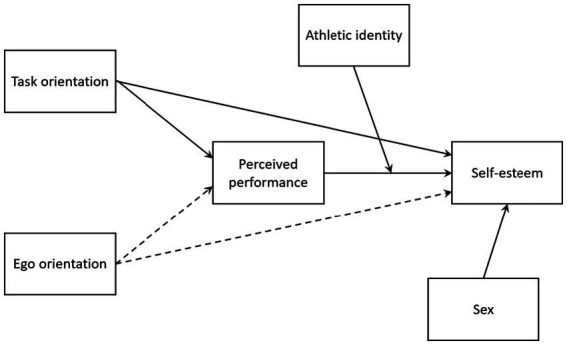
The hypothesized model of the relationship between achievement goal orientations, perceived performance, athletic identity, and self-esteem. The solid lines represent positive relationships, and the dashed lines represent negative relationships. Gender was included in the analysis as a covariate.

The present study aimed to examine how achievement goal orientations relate to self-esteem in student-athletes at lower secondary sport schools, with perceived performance and athletic identity as potential mediator and moderator, respectively (see Figure 1). Based on theoretical postulates and previous research, we hypothesized that:

1. Task goal orientation would be positively related to self-esteem, both directly and indirectly through perceived performance.
2. Ego goal orientation would be negatively related to self-esteem, both directly and indirectly via perceived performance.
3. Athletic identity would moderate the indirect relationship between achievement goal orientations and self-esteem via perceived performance, with the relationship being stronger for athletes with high levels of athletic identity.

## 2 Method

### 2.1 Participants and procedure

This study is part of an ongoing longitudinal and interdisciplinary mixed-methods research project looking at sport schools in Norway, titled ‘Student athlete learning, psychosocial development, mental and physical health, and well-being in secondary school sport classes’, which aims to examine the learning and developmental experiences of young athletes in lower secondary sport schools, as well as the constraints on their pedagogical practice. To determine an appropriate sample size, a multiple regression sample size calculator was used as this is the basis for a conditional process analysis, recommending a minimum of 97 participants to achieve a power level of 0.80 for detecting an anticipated effect size of 0.15 at an alpha level of 0.05, with six variables (Soper, 2018). Using a cross-sectional design, a total of 579 participants (ages 12–16 year; M = 13.93; SD = 0.85) were recruited from seven Norwegian lower secondary sport schools. The sample consisted of 39% female (*n* = 224) and 57% male (*n* = 333) student-athletes, and 4% (*n* = 22) did not want to respond or were classified as “other”.

Ethical approval was granted by The Norwegian Centre of Research Data (reference number 808081) and the University Ethics Committee of the first author’s institution. Informed consent was obtained from all participants and their parents/legal guardians, who were provided with written information about the study. Participants were assured that their responses would remain confidential and accessible only to the research team, not to coaches or school staff. Participation was voluntary, with the option to withdraw at any time without any consequences. A research team member was present during the data collection, which took place during school hours, to answer any questions from participants.

### 2.2 Instruments

#### 2.2.1 Self-esteem

A short version of the Physical Self-Description Questionnaire (PSDQ-S; Marsh et al., 2010) was used to assess participants’ general self-esteem. Participants were asked to reflect on how they had generally felt over the past month, followed by five descriptive statements (e.g., “I have felt that I have much to be proud of”). Responses were rated on a five-point scale ranging from 1 (strongly disagree) to 5 (strongly agree). Higher scores indicate greater self-esteem.

#### 2.2.2 Achievement goal orientation

Participants’ achievement goal orientations were measured based on a scale adapted from Duda and Nicholls (1992). All items were introduced with the stem “I feel really successful in my sport when…” to measure competence standards rather than validation concerns or choice components. Task goal orientation was measured using seven items (e.g., “I do my very best”). Ego goal orientation was measured using six items (e.g., “I’m the only one who can do the skill”). Responses were rated on a five-point scale from 1 (strongly disagree) to 5 (strongly agree). Higher scores indicate stronger task or ego, respectively. Previous research has demonstrated acceptable psychometric properties for this scale in youth sport contexts (Gjesdal et al., 2017).

#### 2.2.3 Perceived performance in sport

The Athlete Satisfaction Questionnaire (ASQ; Riemer and Toon, 2001) was used to assess participants’ perceived satisfaction with their own performance in sport. While the ASQ consists of 15 subscales, this study used the short version, which consists of four-items assessing: (a) absolute performance (e.g., “I’m satisfied with the degree to which I have reached my performance goals during the season”); (b) performance improvements (e.g., “I’m satisfied with the degree of development of my skill level”); and goal achievement (e.g., “I’m satisfied with my goal achievements the last period”). Responses were rated on a seven-point scale from 1 (not at all satisfied) to 7 (extremely satisfied), based on their recent experiences from training and competitions. Higher scores indicate greater perceived performance.

#### 2.2.4 Athletic identity

Participants’ identification with their athletic role was measured using the Athletic Identity Measurement Scale (AIMS; Brewer and Cornelius, 2001). We used seven of the original ten items (e.g., “Sport is the most important part of my life”). We utilized a shortened scale to ensure the questionnaire remained concise, minimizing the burden on participants and encouraging higher response rates, which has been validated for use in the Norwegian sporting context previously (Moen et al., 2019). The scale has been validated for use in the Norwegian sporting context (Moen et al., 2019). Responses were rated on a five-point scale from 1 (strongly disagree) to 5 (strongly agree). Higher scores indicate a stronger identification with the athlete role.

### 2.3 Data analysis

Data were analyzed using IBM SPSS Statistics version 28.0 (Armonk, NY: IBM Corp). A conditional process analysis was conducted to address the study’s objectives (Hayes, 2017). Specifically, we used PROCESS v4.0 macro for SPSS (model 14) to test the direct and indirect (via perceived performance) effects for both task and ego orientation on self-esteem, while including the moderating role of athletic identity in the indirect pathway. Given the documented variation in self-esteem between male and female student-athletes (Saarinen et al., 2025b; Nikander et al., 2022), we included gender as a covariate in the analyses (coding: female and others = 0, male = 1).

PROCESS estimates the indirect effect at different conditional values of the moderator (i.e., athletic identity), testing moderated mediation at three levels of athletic identity: Low (one SD below the mean; moderate (mean level), and high (one *SD* above the mean). If athletic identity moderates the indirect relationship between task and/or ego orientation and self-esteem via perceived performance, the strength and/or direction of this indirect relationship will vary depending on the athletic identity score (Hayes, 2017). To be more robust to non-normal distribution, PROCESS also provide bootstrapping when estimating direct, indirect and conditional indirect effects (Hayes, 2009). We set the bootstrapping at 10,000 samples and bias-corrected 95% confidence intervals (CI) were estimated for all effects. Moderated mediation was considered statistically significant when the 95% CI did not include zero.

## 3 Results

### 3.1 Preliminary analysis

The dataset was examined for missing values and outliers using both multivariate and univariate detection methods (Tabachnick and Fidell, 2013). Little’s Missing Completely at Random (MCAR) test identified missing data for 49 participants (8.6%) and suggested that the data were not missing completely at random (χ^2^ = 163.52, *df* = 1,477, *p* = 0.004). Given that items can be skipped by participants for various reasons (e.g., dealing with sensitive information, having unusual or confusing wording, or an oversight), item-level inspection indicated that missing data were more frequent for items placed at the end of the survey, especially those assessing self-esteem and performance. However, the patterns of missing values did not indicate systemic bias across all variables^1^.

Given the potential impact of missing data on statistical accuracy and interference validity (Schlomer et al., 2010), we employed multiple imputation to handle missing values. Multiple imputation is considered the most effective method for medium sized samples (i.e., 50 < *n* < 1,000) with more than 5% missing data (Cheema, 2014). Using Royston’s (2004) multiple imputation model, five data sets were generated with 100 maximum parameters, and the mean of these data sets was used to replace missing values. Following imputation, 10 cases were identified as multivariate outliers using Mahalanobis distance (*p* < 0.001) and were removed. This is a similar approach used in other studies (e.g., Hurst et al., 2022). Further screening using an inter-quartile threshold of 3.0 did not reveal additional outliers (Sullivan et al., 2021). The final analytical sample consisted of 569 participants.

### 3.2 Descriptive statistics, internal consistency, and zero-order correlations

Descriptive statistics, McDonald’s omega coefficients, and zero-order correlations for all study measures are reported in Table 1. Results indicate that, on average, participants reported scores above the scale’s midpoint for all measures. This suggests that student-athletes’ competence standards reflected both task and ego goal orientations, that they were generally satisfied with their sport performance, identified with their athlete role, and exhibited high self-esteem. Internal consistency was deemed acceptable for all instruments. Zero-order correlations showed that task goal orientation and perceived performance were positively associated with self-esteem, while ego orientation was negatively related. Athletic identity was not significantly associated with self-esteem. However, two AIMS subscales were significantly correlated to self-esteem. That is, social identity was positively related to self-esteem, and negative affectivity was negatively related to self-esteem.

**Table 1:** Descriptive statistics, internal consistency, and zero-order correlations (*N* = 569).

Variable	M (SD)	Omega	1	2	3	4	5	6	7	8	9
1. Task orientation	4.50 (0.45)	0.76	—								
2. Ego orientation	3.65 (0.81)	0.82	0.14^**^	—							
3. Perceived performance	5.22 (1.03)	0.91	0.24^**^	−0.06	—						
4. Athletic identity	4.07 (0.59)	0.75	0.28^**^	0.14^**^	0.18^**^	—					
5. Social identity	4.32 (0.60)	0.61	0.35^**^	0.03	0.30^**^	0.75^**^	—				
6. Exclusivity	3.55 (1.03)	N/A	0.11^**^	0.12^**^	0.12^**^	0.83^**^	0.42^**^	—			
7. Negative affectivity	4.23 (0.76)	N/A	0.19^**^	0.17^**^	−0.04	0.70^**^	0.29^**^	0.40^**^	—		
8. Self-esteem	3.76 (0.66)	0.73	0.24^**^	−0.10^*^	0.59^**^	0.03	0.19^**^	0.01	−0.15^**^	—	
9. Gender	57% male	N/A	−0.18^**^	0.06	0.13^**^	0.03	0.07	0.06	−0.08	0.13^**^	—

Bootstrapped descriptive statistics and zero-order correlation coefficients. Number of bootstrapped resamples = 10,000. The possible range of responses is 1–5 for all variables except perceived performance (1–7). Gender was treated as a dummy variable and coded as 0 = females and others, 1 = males. *p < 0.05; **p < 0.01 (two-tailed).

### 3.3 Main analysis

Aligned with our hypothesized model (see Figure 1), we conducted two moderated mediation analyses to examine the direct and indirect effects of achievement goal orientations on self-esteem, with perceived performance as a mediator and athletic identity as a moderator. To isolate the unique effects of task and ego orientations, each analysis controlled for one predictor while examining the direct effect of the other, ensuring that each achievement orientation’s effect was independent of the other.

**Figure 2:**
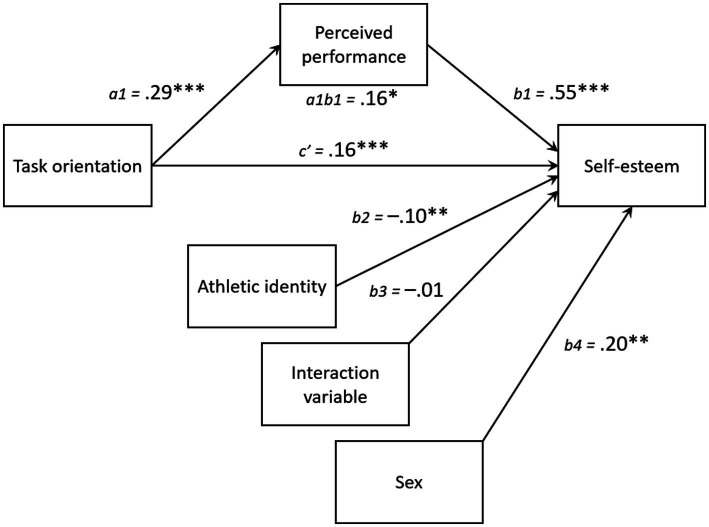
Statistical diagram of the hypothesized moderated mediation model where we propose that the indirect effect of task orientation on self-esteem via perceived performance is moderated by athletic identity. *N* = 569. The values presented are unstandardized bootstrapped regression coefficients. *p < 0.05, **p < 0.01, and ***p < 0.001.

In the first moderated mediation analysis, task orientation was directly related with self-esteem (*b* = 0.16, 95% CI_c’_ = 0.09 to 0.23, *p* < 0.001). Task orientation was also positively associated with perceived performance (*b* = 0.29, 95% CI_a1_ = 0.21 to 0.37, *p* < 0.001), which in turn, independently predicted higher self-esteem (*b* = 0.55, 95% CI_b1_ = 0.48 to 0.62, *p* < 0.001). Perceived performance mediated the relationship between task orientation and self-esteem (*b* = 0.16, 95% CI_a1b1_ = 0.11 to 0.21, *p* < 0.05). Athletic identity was negatively related to self-esteem (*b* = −0.10, 95% CI_b2_ = −0.17 to −0.03, *p* = 0.01). However, the interaction between perceived performance and athletic identity was not significantly associated with self-esteem (*b* = −0.01, 95% CI_b3_ = −0.08 to 0.05, *p* = 0.72), suggesting that athletic identity did not moderate the indirect relationship between task orientation and self-esteem via perceived performance. After controlling for gender, male student-athletes reported greater satisfaction with their performance (*b* = 0.38, 95% CI_a2_ = 0.22 to 0.54, *p* < 0.001) and higher self-esteem (*b* = 0.20, 95% CI_b4_ = 0.06 to 0.33, *p* < 0.01) than female student-athletes (Figure 2).

**Figure 3:**
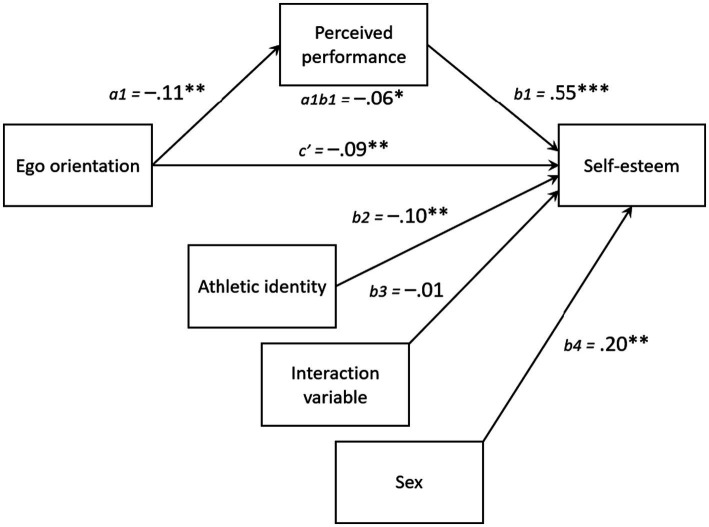
Statistical diagram of the hypothesized moderated mediation model where we propose that the indirect effect of ego orientation on self-esteem via perceived performance is moderated by athletic identity. *N* = 569. The values presented are unstandardized bootstrapped regression coefficients. *p < 0.05, **p < 0.01, and ***p < 0.001.

In the second moderated mediation analysis, ego goal orientation was negatively associated with both self-esteem (*b* = −0.09, 95% CI_c’_ = −0.15 to −0.02, *p* = 0.01) and perceived performance (*b* = −0.11, 95% CI_a1_ = −0.19 to −0.03, *p* < 0.01). Perceived performance again was positively related to self-esteem (*b* = 0.55, 95% CI_b1_ = 0.48 to 0.62, *p* < 0.001) and mediated the relationship between ego orientation and self-esteem (*b* = −0.06, 95% CI_a1b1_ = −0.11 to −0.01, *p* < 0.05). Aligning with the first analysis, athletic identity did not moderate the indirect relationship between ego orientation and self-esteem via perceived performance, as indicated by the interaction variable (*b* = −0.01, 95% CI_b3_ = −0.08 to 0.05, *p* = 0.72). Gender differences also emerged, with male student-athletes reporting greater perceived performance (*b* = 0.38, 95% CI_a2_ = 0.22 to 0.54, *p* < 0.001) and self-esteem (*b* = 0.20, 95% CI_b4_ = 0.06 to 0.33, *p* < 0.01) than their female counterpart (Figure 3).

## 4 Discussion

The study aimed to enhance our understanding of student-athletes’ self-esteem by examining whether achievement goal orientations were directly and indirectly (via perceived performance) related to self-esteem, and whether athletic identity moderated the indirect relationship. Results showed that student-athletes reported high levels of general self-esteem, which aligns with previous studies conducted at the upper secondary sport school level (Nikander et al., 2022). Moreover, the findings generally aligned with expectations, showing that goal orientation was associated with self-esteem, both directly and indirectly through perceived performance. However, contrary to our hypothesis, athletic identity did not moderate this relationship.

### 4.1 Achievement goal orientations, perceived performance and self-esteem

Consistent with prior research (Fox and Lindwall, 2014; Gjesdal et al., 2017), perceived performance emerged as a key factor in the relationship between goal orientations and self-esteem. Specifically, ego goal orientation was negatively associated with self-esteem both directly and indirectly via perceived performance. Task goal orientation was positively associated with self-esteem both directly and indirectly via perceived performance. These results suggest that student-athletes who prioritize personal progress and skill mastery (i.e., task orientation) may experience greater general self-esteem, in part through greater satisfaction with their own performance in sport. In contrast, those who mainly base their competence on outperforming others (i.e., ego orientation) are less satisfied with their performance, which in turn, is linked to lower self-esteem.

The nature of the goal orientations may explain these results. Task goal-oriented athletes rely on self-referenced success criteria, which are more achievable, making it easier to reach them. Conversely, ego goal-oriented athletes depend on external comparisons, making success more difficult to attain, potentially reducing their overall satisfaction as well as self-esteem. Additionally, according to AGT, task goal orientation is also afforded more effort and persistence (Nicholls, 1984; Nicholls, 1989). This should also lead to better performance, especially over time.

The findings underscore the importance of perceived performance in sport schools, and the implications it might have for student-athletes’ more general self-perceptions. This might make them more vulnerable to the pressure to perform that student-athletes often experience from coaches, teachers and significant others (Kavoura and Ryba, 2020; Emrich et al., 2009; Stambulova et al., 2015; Skrubbeltrang et al., 2016). Moreover, Thompson et al. (2022) caution that sport schools may expose student-athletes to inappropriate and unrealistic performance expectations, emphasizing the need for a balanced approach in managing these demands. Our findings reinforce this perspective, adding that a mastery climate should be emphasised when structuring the athletic environment in sport schools.

In line with previous research (Chen et al., 2018), there was a direct link between goal orientations and self-esteem, suggesting that goal orientations is related to general self-esteem beyond its relationship with sport performance. A plausible explanation for the direct link is that goal orientations in sport may reflect a broader achievement mindset. That is, student-athletes who exhibit a strong task or ego orientation in sport may also hold these orientations to academic or personal domains, influencing their general self-esteem. It could also be that achievement goal orientations are related to other health behaviours, that in turn are related to self-esteem. For instance, recent research reported how specific health behaviours, such as diet or sleep patterns were related to self-perceptions in adult student-athletes (Papale et al., 2025). However, this remains speculative and warrants further investigation, particularly considering the orthogonal nature of achievement goal orientations.

### 4.2 Athletic identity and self-esteem

Contrary to our hypothesis and expectations, athletic identity did not moderate the indirect relationship between achievement goal orientations and self-esteem via perceived performance. In fact, athletic identity was not related to self-esteem at all. This aligns with research suggesting that the link between athletic identity and self-esteem is complex. For example, Marin-Urquiza et al. (2018) found no significant relationship between overall athletic identity and self-esteem, but did observe a positive relationship between the social identity component of the AIMS and self-esteem.

In the present study we treated athletic identity as a global factor, in line with Brewer and Cornelius (2001). However, probing the results we see that the social identity subscale of AIMS was positively related to self-esteem, while the negative affectivity was negatively related to self-esteem. As such, identifying as an athlete is not necessarily associated with greater self-esteem. Instead, the nature of the identity, whether it fosters a sense of belonging (i.e., social identity) or pressure or emotional distress (i.e., negative affectivity), may be a more important determinant of self-esteem. For instance, athletes who strongly identify with their athletic role and dedicate significant time and attention to sport may not necessarily experience greater self-esteem than those who maintain a balance between sport and other life domains. This should be examined moving forward, investigating whether there are subdimensions of athletic identity that might be of interest in relation to general self-esteem.

This study highlights the elevated levels of athletic identity observed among student-athletes at the group level. However, the limited variation in the scores likely resulted in no moderating effect detected, as there was probably not enough variance in the moderator. Another explanation concerns the exclusivity dimension of athletic identity, which may have counteracted its potential relationship with self-esteem. High exclusivity scores may indicate a rigid or obsessive commitment to sport, potentially linked to obsessive passion rather than harmonious passion (Vallerand and Verner-Filion, 2020). This may suggest that highly identified athletes may not experience increased self-esteem if their identity is overly contingent on sport success. Thus, it could be that looking at the various subfactors of athletic identity may be a more fruitful avenue when looking at the antecedents of self-esteem among young student-athletes.

### 4.3 Gender differences

A notable finding was the gender differences in perceived performance and self-esteem. Male student-athletes reported higher satisfaction with their sport performance and greater self-esteem compared to female student-athletes. These differences are consistent with previous research on adolescents (Birkeland et al., 2012; Baldwin and Hoffmann, 2002), and a recent study of student-athletes in upper secondary sport schools (Nikander et al., 2022). One possible explanation relates to puberty, which has been linked to declining self-perceptions among female athletes (Monsma et al., 2006). This suggests that sport schools should recognize that maturation may not only affect physical performance but also psychological wellbeing, with gender-specific implications.

Another interpretation concerns self-evaluation biases. It can be assumed that most student-athletes that are accepted to these sport schools are objectively high-performing. As such, this finding might indicate that female student-athletes are more self-critical, leading to lower perceived performance. This aligns with findings that girls tend to underestimate their performance in physical tasks, while boys are more likely to overestimate their abilities (Pesce et al., 2018). Such tendencies may reflect deep-rooted gender norms in sport, including sport schools, influencing how female athletes perceive performance and abilities (Persson, 2023).

### 4.4 Theoretical and practical implications

The present findings extend the existing literature on student-athletes in lower secondary sport schools by identifying key antecedents of their general self-esteem, offering both practical and theoretical insights. From a theoretical perspective, the results underscore the applicability of AGT in understanding the psychological outcomes of student-athletes, particularly the role of task and ego orientations in relation to self-esteem and perceived performance. The findings highlight the notion that self-referenced criteria are more achievable and psychologically beneficial than external comparisons, as suggested by AGT (Nicholls, 1984, 1989). Furthermore, the gender disparities observed in perceived performance and self-esteem align with theories of self-evaluation biases and sociocultural influences on gendered perceptions of competence in sport. These insights contribute to a deeper understanding of how gender norms and developmental factors may uniquely shape the self-perceptions of male and female student-athletes.

These theoretical implications lead to several practical recommendations for educators and coaches. A key takeaway is the importance of fostering task-oriented achievement goals among student-athletes. Task-oriented athletes experience higher self-esteem and greater satisfaction with their progress, making interventions aimed at reinforcing self-referenced success criteria particularly valuable. For sport schools aiming to enhance their students’ self-perceptions beyond the sporting context, implementing structured programs that promote task-oriented goal setting could be beneficial. For example, research in upper secondary sport schools has shown that affective coaching styles are positively related to task goal orientation (Saarinen et al., 2024). Integrating coaching strategies that emphasize effort, personal growth, and mastery may, therefore, help student-athletes develop a healthier mindset toward performance and self-worth.

Additionally, the gender differences observed in this study suggest that female student-athletes may experience greater self-critical tendencies and lower self-esteem, despite likely comparable objective performance levels. This highlights the need for gender-sensitive interventions tailored to address these psychological disparities. From a theoretical standpoint, these findings suggest that self-evaluation biases, rooted in gendered socialization and norms in sport, may negatively impact female athletes’ psychological well-being. Practical solutions could include implementing integrated psychological skills training programs designed specifically for female student-athletes, aimed at building confidence and reducing self-critical tendencies. Recent research has shown promising results from such interventions (Merlin et al., 2024). By addressing both the theoretical and practical dimensions of self-esteem development, sport schools can create a more supportive and inclusive environment for all student-athletes.

### 4.5 Limitations and future research

While the study provides valuable insights, several limitations should be considered when interpreting the findings. First, the cross-sectional design limits the ability to establish causal relationships between the variables. Future research would benefit from a longitudinal design to track how goal orientations, perceived performance, and self-esteem evolve over time. Second, as the Little’s MCAR test indicated that data were not missing completely at random, some caution is warranted when interpreting the results, particularly those related to self-esteem and perceived performance. Third, the study sample reported high scores across most constructs, with limited variability in responses. This homogeneity, combined with a small sample size, may have reduced the ability to detect nuanced effects, particularly in the moderating role of athletic identity. Additionally, the use of a shortened version of the AIMS may also have contributed. Future studies should aim for larger and more diverse samples to explore potential contextual and individual differences in these relationships, and to conduct the analyses using more rigorous methods such as structural equation modelling.

## 5 Conclusion

This study examined the relationship between achievement goal orientations, perceived performance, athletic identity and self-esteem among student-athletes in lower secondary sport schools. These findings bring important insights to why some young athletes experience more positive self-esteem than others and reinforce the importance of promoting a task-oriented climate in sport schools. By encouraging self-referenced success criteria, supporting gender-specific self-esteem interventions, and implementing affective coaching strategies, sport schools can play a vital role in fostering psychological well-being and personal development among student-athletes.

## Data Availability

The raw data supporting the conclusions of this article will be made available by the authors, without undue reservation.

## Footnotes

1 Item-level missingness ranged from 0.3 to 8.5%, with higher missingness observed for items pertaining to self-esteem (7.1 to 8.5% missing), performance (2.9 to 6.6% missing), and athletic identity (2.8 to 3.8% missing) compared to items concerning task orientation (0.3 to 1.4% missing) and ego orientation (1.4 to 3.1% missing).
